# Cardiac Specific Overexpression of Mitochondrial Omi/HtrA2 Induces Myocardial Apoptosis and Cardiac Dysfunction

**DOI:** 10.1038/srep37927

**Published:** 2016-12-07

**Authors:** Ke Wang, Yuexing Yuan, Xin Liu, Wayne Bond Lau, Lin Zuo, Xiaoliang Wang, Lu Ma, Kun Jiao, Jianyu Shang, Wen Wang, Xinliang Ma, Huirong Liu

**Affiliations:** 1 Department of Physiology and Pathophysiology, School of Basic Medical Sciences, Capital Medical University, Beijing, 100069, P. R. China; 2 Department of Emergency Medicine, Thomas Jefferson University, 1020 Sansom Street, Philadelphia, PA 19107, USA; 3 Department of Physiology, Shanxi Medical University, Taiyuan, Shanxi, 030001, P. R. China; 4 Beijing Key Laboratory of Metabolic Disturbance Related Cardiovascular Disease, Capital Medical University, Beijing, 100069, P. R. China

## Abstract

Myocardial apoptosis is a significant problem underlying ischemic heart disease. We previously reported significantly elevated expression of cytoplasmic Omi/HtrA2, triggers cardiomyocytes apoptosis. However, whether increased Omi/HtrA2 within mitochondria itself influences myocardial survival *in vivo* is unknown. We aim to observe the effects of mitochondria-specific, not cytoplasmic, Omi/HtrA2 on myocardial apoptosis and cardiac function. Transgenic mice overexpressing cardiac-specific mitochondrial Omi/HtrA2 were generated and they had increased myocardial apoptosis, decreased systolic and diastolic function, and decreased left ventricular remodeling. Transiently or stably overexpression of mitochondria Omi/HtrA2 in H9C2 cells enhance apoptosis as evidenced by elevated caspase-3, -9 activity and TUNEL staining, which was completely blocked by Ucf-101, a specific Omi/HtrA2 inhibitor. Mechanistic studies revealed mitochondrial Omi/HtrA2 overexpression degraded the mitochondrial anti-apoptotic protein HAX-1, an effect attenuated by Ucf-101. Additionally, transfected cells overexpressing mitochondrial Omi/HtrA2 were more sensitive to hypoxia and reoxygenation (H/R) induced apoptosis. Cyclosporine A (CsA), a mitochondrial permeability transition inhibitor, blocked translocation of Omi/HtrA2 from mitochondrial to cytoplasm, and protected transfected cells incompletely against H/R-induced caspase-3 activation. We report *in vitro* and *in vivo* overexpression of mitochondrial Omi/HtrA2 induces cardiac apoptosis and dysfunction. Thus, strategies to directly inhibit Omi/HtrA2 or its cytosolic translocation from mitochondria may protect against heart injury.

Cardiovascular disease is the leading cause of death world-wide. The World Health Organization (WHO) estimates that approximately 20 million cardiovascular diseases (CVD) related deaths will occur in 2015^1^,^2^. Substantial evidence suggests that cardiomyocyte apoptosis is a major contributor to CVD and aging-related cardiac dysfunction^3^ and occurs with ischemia/reperfusion injury^4^ and with dilated cardiomyopathy^5^. Although mitochondrial impairment plays a pivotal role in cardiomyocyte apoptosis^6^, the specific underlying molecular mechanisms remain unknown.

Omi/HtrA2 is a proapoptotic mitochondrial serine protease that is released into the cytoplasm following apoptotic insult^7^. It complexes with different inhibitors-of-apoptosis proteins (IAPs), preventing their ability to bind and attenuate caspases^8^. We previously reported that myocardial ischemia/reperfusion (MI/R) resulted in the translocation of Omi/HtrA2 from the mitochondria to the cytoplasm, promoting cardiomyocyte apoptosis^9^. In addition, we observed that increased expression of Omi/HtrA2 in aging rats augmented MI/R injury by again stimulating myocardial apoptosis^10^. Subsequent studies have confirmed similar results regarding translocation of Omi/HtrA2 in both myocardial and cerebral ischemia/reperfusion models^11^,^12^. Recent evidence suggests that Omi/HtrA2 has unique a pro-apoptotic function within the mitochondria^13^, and that in human neutrophils exposed to TNF-α, Omi/HtrA2 induces apoptosis without ever being released from the mitochondria^14^. Whether mitochondrial Omi/HtrA2 may be involved with *in vivo* cardiomyocyte apoptosis to relevant degree has never been determined.

Therefore, the aims of the present study were (1) to determine the effect of cardiac specific overexpression of intra-mitochondrial Omi/HtrA2 on cardiac structure and function; (2) to ascertain whether intra-mitochondrial Omi/HtrA2 directly promotes cardiomyocyte apoptosis, and to investigate the possible mechanisms.

## Results

### Cardiomyocytes apoptosis, cardiac dysfunction, and left ventricular remodeling in transgenic mice overexpressing cardiac Omi/HtrA2

**Figure 1:**
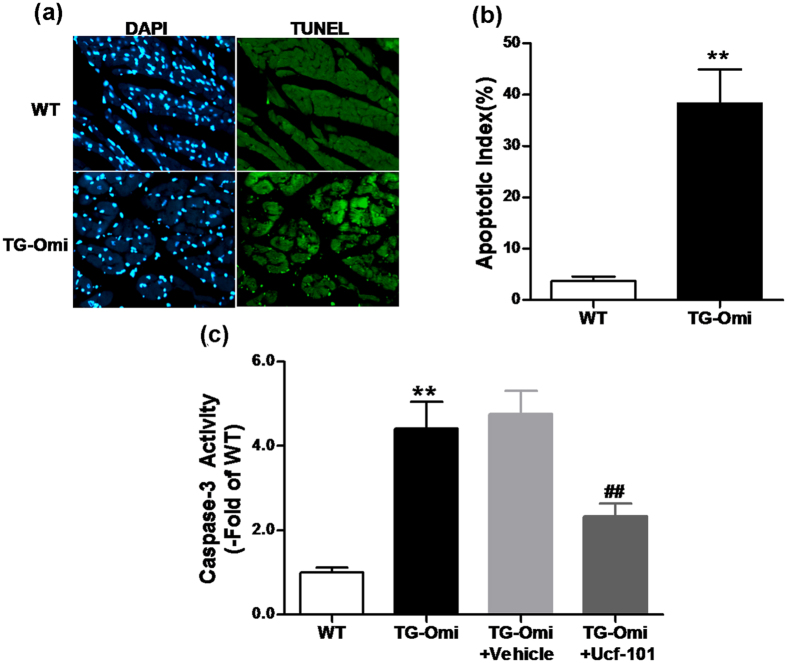
Cardiac-specific overexpression of Omi/HtrA2 induced cardiomyocyte apoptosis. (**a**) TUNEL labeling. (**b**) Apoptotic index, assessed by measuring the percentage of apoptotic nuclei from total nuclei. (**c**) Caspase-3 activity in WT and transgenic mice with cardiac-specific overexpression of Omi/HtrA2 (TG-Omi). TG-Omi was treated with DMSO (Vehicle), Ucf-101 (Omi/HtrA2 inhibitor). n = 3–5 /genotype. **p < 0.01 versus WT. ^##^*p* < 0.05 versus TG-Omi + Vehicle.

To measure effects of augmented Omi/HtrA2 *in vivo*, we used a transgenic mouse model that overexpressed cardiac-specific Omi/HtrA2 (Saiye Biotech Limited Company) as confirmed by western blot and RT-PCR (Supplementary Figure 1). Effects of cardiac-specific overexpression of Omi/HtrA2 on myocardial tissue were assessed with TUNEL staining of myocardial tissue sections. TUNEL-positive nuclei in transgenic mice was increased around 10-fold over that in wild type (WT) mice (38.3 ± 6.7 *vs.* 3.7 ± 0.9, *p* < 0.01, Fig. 1a,b). Also, caspase-3 activity in cardiomyocytes of mice overexpressing Omi/HtrA2 was increased 4-fold over that in WT tissue and this was attenuated by Ucf-101, an Omi/HtrA2 specific inhibitor (Fig. 1c).

**Figure 2:**
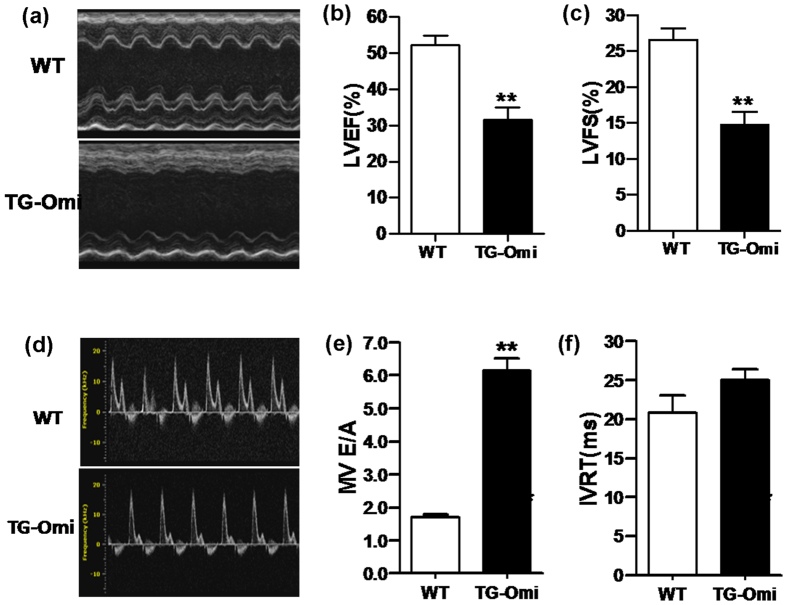
Cardiac dysfunction in transgenic mice with cardiac-specific overexpression of Omi/HtrA2. (**a**) Echocardiography of WT and transgenic mice overexpressing cardiac-specific Omi/HtrA2 (TG-Omi). (**b**) EF (ejection fraction), %. (**c**) FS (fractional shortening), %. (**d**) Doppler imaging of the mitral annulus. (**e**) E/A ratio, the ratio of the early (E) to late (A) ventricular filling velocities. (**f**) IVRT (isovolumetric relaxation time). All indices were recorded at 9 months age. n = 3–5 /genotype. **p < 0.01 versus WT.

To identify effects of cardiac specific overexpression of intra-mitochondrial Omi/HtrA2 upon cardiac function and structure, left ventricle systolic function was evaluated via echocardiography (Fig. 2a). At 6 months-of-age, Omi/HtrA2 transgenic mice had cardiac systolic dysfunction, as evidenced by a significantly decreased ejection fraction (Fig. 2b) and fractional shortening (Fig. 2c), compared to age-matched WT controls at baseline. Doppler imaging of the mitral annulus, to evaluate cardiac diastolic function (Fig. 2d), indicated that the E/A ratio (ratio of the early (E) to late (A) ventricular filling velocities) of transgenic mice was significantly greater compared to WT mice (Fig. 2e), although there was no difference in isovolumetric relaxation time (IVRT) between transgenic and WT mice (Fig. 2f). Thus, cardiac-specific Omi/HtrA2 overexpression impaired both systolic and diastolic function.

**Figure 3:**
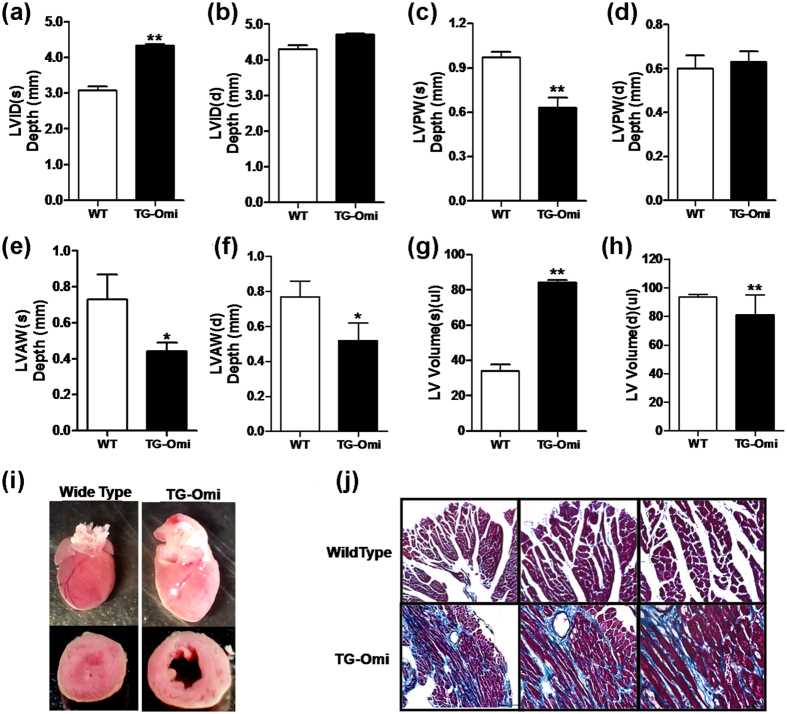
Cardiac remodeling in transgenic mice overexpressing cardiac-specific Omi/HtrA2. (**a**) Left ventricular end-systolic diameter (LVID(s), mm). (**b**) Left ventricular end-diastolic diameter (LVID(d), mm). (**c**) Left ventricular posterior wall end-systolic thickness (LVPW(s), mm). (**d**) Left ventricular posterior wall end-diastolic thickness (LVPW(d), mm). (**e**) Left ventricular anterior wall end-systolic thickness (LVAW(s), mm). (**f**) Left ventricular anterior wall end-diastolic thickness (LVAW(d), mm). (**g**) Left ventricular volume during systole period (LV volume(s), l). (**h**) Left ventricular volume during diastole period (LV volume (d),(l). (**i**) Gross pathology of enlarged ventricles in Omi/HtrA2 transgenic mice compared to WT littermates, 3 months-of-age. (**j**) Omi/HtrA2 transgenic mice had more fibrosis (blue trichromestain) than WT, 9 months-of-age. n = 3–5/genotype. *p < 0.05, **p < 0.01 versus WT.

To understand how cardiac-specific overexpression of Omi/HtrA2 altered cardiac structure, we assessed cardiac morphology using echocardiography in transgenic and WT mice that were 9 months-of-age. Figure 3 depicts echocardiography that documents increased left ventricular end-systolic diameter (LVID (s)) (Fig. 3a), decreased left ventricular posterior wall end-systolic thickness (LVPW(s)) (Fig. 3c), and decreased left ventricular wall end-systolic anterior thickness (LVAW(s)) (Fig. 3e) and left ventricular wall end-diastolic anterior thickness (LVAW (d)) (Fig. 3f) in the mice overexpressing cardiac-specific Omi/HtrA2. Increased LV volume was observed during both systole and diastole in transgenic mice (Fig. 3g,h). No significant differences were noted in LVID (d) or LVPW (d) between transgenic and WT mice (Fig. 3b,d). Histology revealed that mice with cardiac-specific Omi/HtrA2 overexpression had enlarged left ventricles with thinner ventricular walls (Fig. 3i), which is consistent with the echocardiographic data and with dilated cardiomyopathy. Furthermore, Masson staining revealed significantly increased peripheral collagen surrounding cardiomyocytes in transgenic mice compared to WT (Fig. 3j).

### Overexpression of mitochondrial Omi/HtrA2 directly increases apoptosis

**Figure 4:**
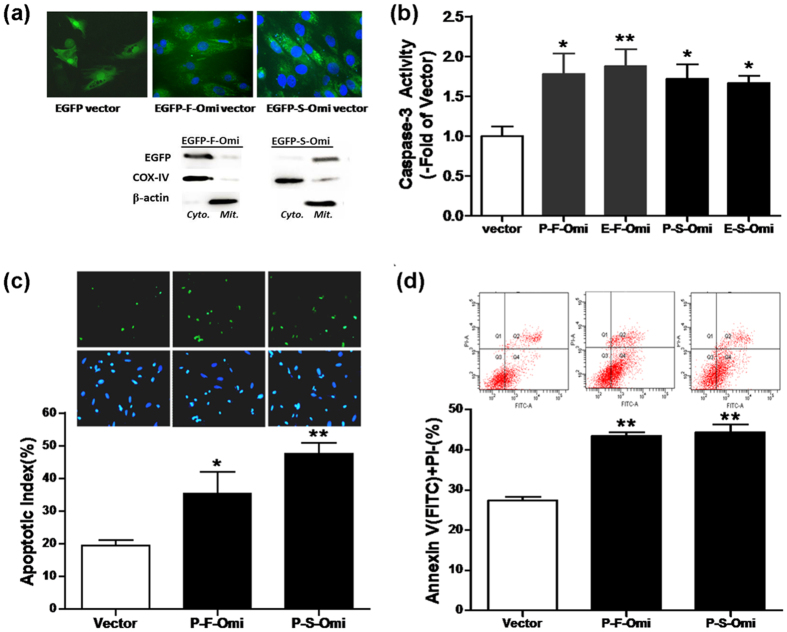
Increased apoptosis in H9C2 cells transfected with either mitochondrial Omi/HtrA2 or cytosolic Omi/HtrA2. (**a**) Fluorescent images and Omi/HtrA2 protein in either mitochondrial (mito.) or cytosolic (cyto.) fraction in H9C2 cells transfected with both mitochondrial Omi/HtrA2 and cytosolic Omi/HtrA2. (**b**) Caspase-3 activity. (**c**) TUNEL labeling (up) and apoptotic index (down). (**d**) Annexin V and PI staining. Vector: PcDNA3.1 or EGFP vector; P-F-Omi or E-F-Omi: Pc3.1 vector or EGFP vector with mitochondrial Omi/HtrA2; P-S-Omi or E-S-Omi: Pc3.1 vector or EGFP vector with cytosolic Omi/HtrA2; Full-Omi, Pc3.1 vector with mitochondrial Omi/HtrA2; Short-Omi, Pc3.1 vector with cytosolic Omi/HtrA2. n = 10–12 each. *p < 0.05, **p < 0.01 versus Vector.

To establish the effect of different sources of Omi/HtrA2 overexpression in cardiomyocytes, H9C2 cells were transiently transfected with either mitochondrial (with mitochondrial targeting sequence, MTS) or cytosolic (without MTS) Omi/HtrA2 (Fig. 4a). Overexpression of mitochondrial or cytosolic Omi/HtrA2 increased H9C2 cellular apoptosis, as evidenced by increased caspase-3 activity (Fig. 4b), increased TUNEL-positive nuclei (Fig. 4c), and increased positive Annexin V-FITC staining for phosphatidylserine translocation (Fig. 4d,e).

**Figure 5:**
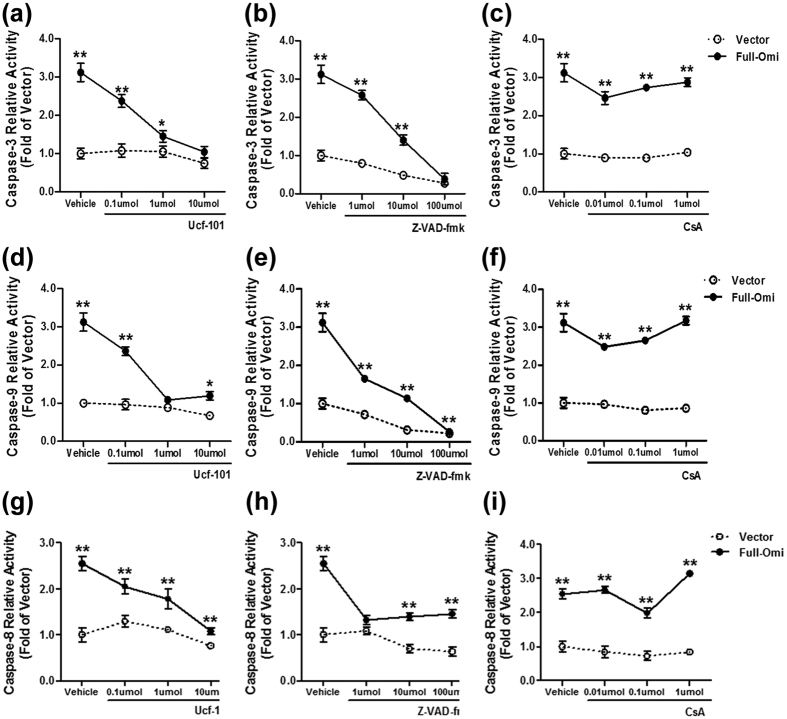
Overexpression of mitochondrial Omi/HtrA2 increased apoptosis. (**a–c**) Caspase-3 activity. (**d–f**) Caspase-9 activity. (**g–i**) Caspase-8 activity. Both eGFP (Vector)- and mitochondrial Omi/HtrA2 (Full-Omi)-transfected H9C2 cell lines were treated with varying doses of DMSO (Vehicle), Ucf-101 (Omi/HtrA2 inhibitor), Z-VAD-FMK (caspase inhibitor), or CsA (mitochondrial permeability transition pore inhibitor) for 24 h before measuring Caspase-3 (**a–c**), Caspase-9 (**d–f**) and -8 (**g–i**). n = 10–12 each. *p < 0.05, **p < 0.01 versus Vector.

To confirm the above observation that increased apoptosis related to mitochondrial overexpression of Omi/HtrA2, a H9C2 cell line stably overexpressing mitochondrial Omi/HtrA2 was purchased and identified by immunoblot or fluorescent microscopy (Supplementary Figure 2A). Both Omi/HtrA2 mRNA (Supplementary Figure 2B) and protein (Supplementary Figure 2C) were increased in H9C2 cells stably overexpressing mitochondrial Omi/HtrA2. Both the mitochondrial and cytosolic fractions of H9C2 cells were isolated separately and incubated with Omi/HtrA2 antibody. No cytoplasmic Omi/HtrA2 was expressed in the transfected H9C2 cell line (Supplementary Figure 2D). Nevertheless, mitochondrial overexpression of Omi/HtrA2 increased apoptosis as evidenced by increased caspase-3, caspase-8 and caspase-9 activity, and all of this was attenuated by incubating cells with either Ucf-101 (an Omi/HtrA2 specific inhibitor) or Z-VAD-FMK (a caspase inhibitor), but not cyclosporine A (CsA, a mitochondrial permeability transition pore inhibitor, Fig. 5a–i).

### Overexpression of mitochondrial Omi/HtrA2 increased apoptosis, and this may be related to HAX-1 degradation

**Figure 6:**
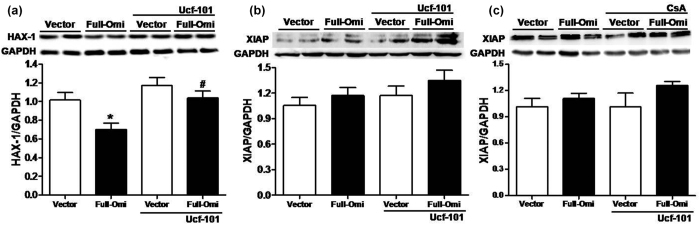
(**a**) Effect of Ucf-101 on HAX-1 expression in H9C2 cells overexpressing mitochondrial Omi/HtrA2. Effect of either Ucf-101 (**b**) of CsA (**c**) upon the XIAP expression in H9C2 cells overexpressing mitochondrial Omi/HtrA2. Both the H9C2 cell line stably with eGFP (Vector) and H9C2 cell line stably with mitochondrial Omi/HtrA2 (Full-Omi) were treated with either 0.1uM Ucf-101(Omi/HtrA2 inhibitor) or 0.1 μM cyclosporine A (CsA, mitochondrial permeability transition pore inhibitor) for 24 hours. Representative Western blots showing cytosolic XIAP or HAX-1 expression. GAPDH was used as a loading control. n = 6 each. *p < 0.05 versus Vector; ^#^*p* < 0.05 versus Full-Omi.

In 2004, Cilenti *et al*.^15^ identified HS1-associated protein X-1 (HAX-1) as a specific binding partner to Omi/HtrA2 and subsequent studies determined it to be an anti-apoptotic protein that inhibits caspases-3 and -9 activation^16^. This suggests that HAX-1 may be involved in mitochondrial Omi/HtrA2-induced cardiomyocyte apoptosis. However, whether overexpression of mitochondrial Omi/HtrA2 overwhelms HAX-1, thereby shifting the balance towards unmitigated cardiomyocyte apoptosis is unknown. To confirm involvement of HAX-1 in the underlying mechanisms responsible for mitochondrial Omi/HtrA2 overexpression-mediated apoptosis, HAX-1 expression was measured in the H9C2 cell line stably overexpressing mitochondrial Omi/HtrA2. Indeed, overexpression of mitochondrial Omi/HtrA2 decreased HAX-1 expression and this effect was attenuated by Ucf-101 administration (Fig. 6a). Omi/HtrA2 inactivates XIAP irreversibly and facilitates caspase activity in the cytoplasm^8^. However, in H9C2 cells stably overexpressing mitochondrial Omi/HtrA2, XIAP expression was not affected and neither Ucf-101 nor CsA treatment altered XIAP expression (Fig. 6b,c).

### Overexpression of mitochondrial Omi/HtrA2 rendered H9C2 cells more susceptible to hypoxia/reoxygenation injury

**Figure 7:**
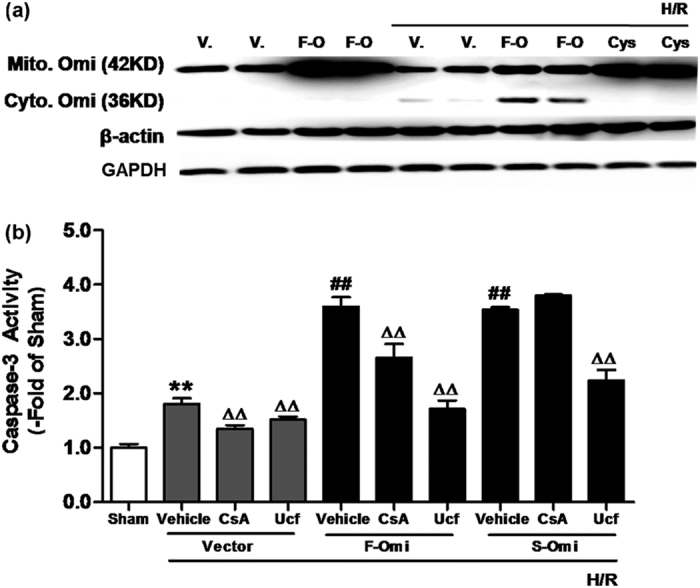
Cyclosporine A treatment of H9C2 cells transfected with either mitochondrial Omi/HtrA2 or cytosolic Omi/HtrA2 after hypoxia and reoxygenation (H/R). (**a**) Cyclosporine A (CsA, 0.1μmol) completely blocked translocation of Omi/HtrA2 from mitochondria (42 kD, mitochondrial Omi/HtrA2) to cytoplasm (36 kD, cytosolic Omi/HtrA2) after H/R. (**b**) CsA inhibited mitochondrial Omi/HtrA2-induced activation of caspase-3 after H/R. Vector (V): Pc3.1 vector; F-Omi (F-O): Pc3.1 vector with mitochondrial Omi/HtrA2; S-Omi: Pc3.1 vector with cytosolic Omi/HtrA2. H9C2 cells were treated with vehicle (DMSO) and cyclosporine A (CsA), a mitochondrial permeability transition pore inhibitor. n = 10–12 each. **p < 0.01 versus Sham; ^##^*p* < 0.01 versus Vector + H/R; ^∆∆^*p* < 0.01 versus Vehicle.

To investigate the functional consequence of increased mitochondrial Omi/HtrA2, we employed an *in vitro* model of simulated ischemia/reperfusion (I/R) injury. Cultured H9C2 cells were subjected to 24 hours hypoxia (H), followed by 3 hours reoxygenation (R). H/R induced increased cytoplasmic translocation of Omi/HtrA2 from the mitochondria, evidenced by western analysis (increased density of the 36KD cytosolic Omi/HtrA2 band in transfected cells compared to control, Fig. 7a). Plasmid-transfected H9C2 cells stably overexpressing mitochondrial Omi/HtrA2 exhibited increased apoptosis after H/R, evidenced by increased caspase-3 activity (Fig. 7b). To further investigate the cytoplasmic translocation of mitochondrial Omi/HtrA2 after H/R, cells were treated with cyclosporine A (CsA), a mitochondrial permeability transition pore inhibitor. After CsA treatment, no 36KD Omi/HtrA2 band was observed, suggesting CsA blocked the cytoplasmic translocation of Omi/HtrA2 from the mitochondria (Fig. 7a). CsA administration decreased H/R-induced apoptosis in transgenic H9C2 cells overexpressing mitochondrial Omi/HtrA2, but not cytosolic Omi/HtrA2. However, CsA conferred less protection against H/R induced apoptosis compared to Ucf-101 (an inhibitor of Omi/HtrA2, Fig. 7b). Together, these results suggest increased mitochondrial Omi/HtrA2 not only increases its translocation to the cytoplasm, but also directly increased myocardial apoptosis after H/R.

## Discussion

Myocardial morphometric and functional observations from the present study indicate that cardiac-specific overexpression of Omi/HtrA2 in the mitochondria significantly induced myocardial apoptosis, cardiac remodeling, and cardiac dysfunction that resembles dilated cardiomyopathy. Transgenic H9C2 cells overexpressing mitochondrial or cytosolic Omi/HtrA2 had increased apoptosis under basal conditions, an effect reversed partly by the mitochondrial permeability transition pore inhibitor CsA. Furthermore, H9C2 cells overexpressing mitochondrial Omi/HtrA2 are significantly more sensitive to H/R-induced apoptosis, an effect blocked by the mitochondrial permeability transition pore inhibitor CsA. These results not only support our previous study suggesting that cytoplasmic translocation of Omi/HtrA2 from the mitochondria promoted cardiomyocyte apoptosis after myocardial infarction (MI)^9^, a common translocational mechanism of Omi/HtrA2 induced apoptosis, but also yield mechanistic insight regarding how mitochondrial Omi/HtrA2 directly promotes myocardial apoptosis.

Our and other’s previous studies showed the expression of Omi/HtrA2 is increased in the aging heart, which promotes cardiomyocyte apoptosis via degradation of XIAP and plays a causative role in enhanced post-ischemic injury in the aging heart^10^,^17^. Here, we confirmed these findings using a transgenic model of mitochondrial Omi/HtrA2 overexpression that mimicked the conditions of an aged heart. Consistent with our prior reports, an H9C2 cell line stably overexpressing mitochondrial Omi/HtrA2 had enhanced Omi/HtrA2 cytosolic leakage and apoptotic death after H/R. The pro-apoptotic role of Omi/HtrA2 in pathology has been investigated extensively and although mechanisms underlying deleterious effects of Omi/HtrA2 translocation and degradation XIAP have been identified^9^,^10^,^11^,^13^. However, studies suggest that the role of Omi/HtrA2 is complex^13^,^18^,^19^. Ischemia-induced myocardial cell death was also mediated by the mitochondria-derived reactive oxygen species (ROS). It has been reported that the Omi/HtrA2 alter the ROS system^20^,^21^, like inhibition for instance MnSOD^14^, which might be involved in MI/R injury. Besides of apoptosis, the cytosolic Omi/HtrA2 release from mitochondria can also contribute to another cell death phenotype like anoikis via its protease activity in intestinal epithelial cells^22^,^23^. It has been shown that Omi/HtrA2-induced anoikis might be related to the cleavage of cytoskeleton-associated proteins through the modulation of mitochondrial dynamics. Proteomic analysis indicated that vimentin, actin, α- and β-tubulin are substrates for Omi/HtrA2 protease activity^24^. Studies reported Ras transformation, accumulation of p53 resulted in the release of Omi/HtrA2 into cytosol via disruption of mitochondrial integrity, where it induces actin cytoskeleton disassembly^25^ and vimentin cleavage^26^. Therefore, the translocation of Omi/HtrA2 into cytosol would be involved in many cell death phenotypes. In our previous^9^,^10^ and current studies, either ischemia/reperfusion (I/R) or hypoxia/reoxygenation (H/R) induced increased cytoplasmic translocation of Omi/HtrA2 from the mitochondria, which enhanced the myocardial apoptosis. Although there was no evidence to show the anoikis was directly contributed to the myocardial ischemia/reperfusion injury, the hypothesis of cytoplasmic translocation of Omi/HtrA2-induced the anoikis and its mechanisms in cardiomyocytes after I/R would be worth to investigate in the future study.

In the current study, interestingly, enhanced apoptosis was observed in transgenic cells overexpressing mitochondrial Omi/HtrA2 could not be completely blocked via inhibition of cytosolic Omi/HtrA2 translocation from the mitochondria. This important result suggests the existence of a non-translocational mechanism underlying Omi/HtrA2-mediated apoptosis. Omi/HtrA2 is a nuclear-encoded mitochondrial serine protease with pro-apoptotic function in mammalian cells^27^. Upon induction of apoptosis, Omi/HtrA2 translocates to the cytoplasm where it participates in regulating caspase-dependent apoptosis by binding and degrading inhibitors of apoptosis, such as XIAP^15^. Omi/HtrA2 has been proposed to enhance caspase activation via multiple pathways other than cytosolic translocation^19^, and Blink *et al*.^14^ reported that Omi/HtrA2 can induce apoptosis without mitochondrial release. We provided the first evidence that overexpression of mitochondrial Omi/HtrA2 directly promotes cardiomyocytes apoptosis *in vivo*. Recent studies support a distinct function of Omi/HtrA2 in maintaining mitochondrial homeostasis^19^ by functioning as a chaperone^28^. A single mutation in the Omi/HtrA2 gene (mnd2) in mice causes a phenotype associated with muscle wasting, neurodegenerative disease, and death by 6 weeks-of-age^29^. However, many apoptotic proteins, such as cytochrome c and endonuclease G, with other mitochondrial functions play distinctly different roles during apoptosis^15^,^30^. In a similar fashion, Omi/HtrA2 has a “chaperone-like” function in the mitochondria, as well as a pro-apoptotic role.

Omi/HtrA2 may regulate apoptosis at a pre-mitochondrial level, ultimately activating caspase-3 and -9. We use the CsA to block the opening of mitochondrial permeability transition pore (mPTP). The binding of CsA to cyclophilin-D results in the inhibition of mPTP opening, which prevents collapse of membrane potential, uncoupling of the respiratory chain, mitochondrial disruption and the release of cytochrome c as well as other proapoptotic factors^31^. Our results demonstrated that CsA (mitochondrial permeability transition pore inhibitor cyclosporine A) administration did not decrease XIAP and reverse apoptosis, thereby suggesting mitochondrial Omi/HtrA2 may induce apoptosis without translocating from mitochondria. Additionally, Omi/HtrA2 might regulate the mitochondrial morphology and function through interaction with its substrates in mitochondria, such as LON protease 1^32^,^33^, MnSOD^14^, OPA-1^34^ and Mpv17l^35^ protein family. Therefore, the mechanisms of mitochondrial Omi/HtrA2 induced mitochondrial dysfunction would be worth to investigate in the future study.

We found mitochondrial Omi/HtrA2 overexpression increased cardiomyocyte apoptosis, independent of its release from the mitochondria, which might be related to the degradation of the anti-apoptotic mitochondrial molecule HAX-1. In several previous studies^15^,^16^,^36^, co-immunoprecipitaion with Omi/HtrA2 and HAX-1 demonstrated Omi/HtrA2 induced apoptosis by cleavage with HAX-1 protein. HAX-1 exerts anti-apoptotic effects through different pathways, either via inhibition of caspase-9 and -3 activation, or by modulating Ca2^+^ homeostasis via direct interaction with two major sarcoplasmic reticular proteins (the Ca^2+^ ATP-ase pump and its regulator phospholamban)^36^,^37^. HAX-1, as one of the proteins that interact with caspase-9, is highly expressed in the heart. Knock down of HAX-1 gene products demonstrated the essential function of HAX-1 in cardiomyocytes survival^38^. Most importantly, the heart is characterized by a high level of caspase-9 expression^38^. In our study, overexpression of Omi/HtrA2 induces the degradation of HAX-1 and increased caspase-9 activity without apoptotic stimuli, and this effect was attenuated by Omi/HtrA2 inhibitor Ucf-101 administration, indicating HAX-1 might better elucidate the molecular mechanism of pro-apoptotic effects of Omi/HtrA2 in heart. Concurrently, we got the similar results from our *in vivo* transgenic mice model. (Supplementary Figure 1E). All these results supported our conclusion that the mitochondrial Omi/HtrA2 promotes cardiomyocytes apoptosis again. However, the exact mechanism by which HAX-1 is degraded by Omi/HtrA2 during ischemia/reperfusion injury is the subject of ongoing investigation, although recent evidence suggests that HAX-1 can either function as an anti- or pro-apoptotic regulator, and in turn influence cell survival or death, through homo- or heterodimerization^39^. Future studies are therefore needed to determine whether Omi/HtrA2 helps balance the formation of HAX-1 homo- or heterodimers in cardiomyocytes.

All of our above results and other studies suggested that overexpressing mitochondrial Omi/HtrA2 *in vitro* results in apoptotic cell death^14^,^40^. Meanwhile, in our *in vivo* model, transgenic mice with cardiac-specific overexpression of mitochondrial Omi/HtrA2 not only promoted cardiomyocyte apoptosis, but also exhibited cardiac dysfunction (abnormal ejection fraction and fractional shortening). Cardiomyocytes loss due to apoptosis is recognized as an important determinant of structure and function of the heart^41^. The decreased LVAW and LVPW and increased LVID observed in transgenic mice overexpressing mitochondrial Omi/HtrA2 suggested enlarged left ventricles with thinned ventricular walls. Increased infiltrating collagenous content was evident upon histologic analysis. Taken together, these results strongly suggest cardiac-specific mitochondrial overexpression of Omi/HtrA2 exacerbates cardiac injury *in vivo*.

In summary, we report that overexpression of mitochondrial Omi/HtrA2 exacerbates cardiac apoptosis and contractile dysfunction *in vivo*; suggesting strategies to directly inhibit Omi/HtrA2 within the mitochondria and to preserve mitochondrial membrane integrity may have promise for reducing cardiomyocyte apoptosis in various cardiac diseases.

### Limitations

In our studies, the H9C2 cell line was used for *in vitro* studies. Although experiments with isolated adult cardiomyocytes would be best, these cells are difficult to transfect and cannot be manipulated to stably overexpress mitochondrial Omi/HtrA2. The current studies are nevertheless suggestive of alternative mechanisms and are supported by the *in vivo* studies. They also provide the necessary preliminary data to investigate these mechanisms further, possibly using primary cells, and can even be used with *in vitro* drug screens.

## Materials and Methods

### Animals

All mouse experiments were performed in strict accordance with the “Guiding Principles in the Use and Care of Animals” published by the National Institutes of Health (NIH Publication No. 85-23, Revised 1996) and were approved by the Institutional Animal Care and Use Committee of Capital Medical University. Three-month-old wild type (WT) mice and cardiac Omi/HtrA2-overexpressing mice were purchased from the Saiye Biotech Limited Company (Guangzhou, China).

### Omi Gene clone and plasmid construct

Total RNA was extracted from H9C2 cells and purified using RNeasy Mini Kit (Qiagen TM, Valencia, CA), according to the manufacturer’s instructions. For cDNA synthesis, 5 μg of total RNA samples were reverse-transcripted using SuperScript^®^ III First-Strand Synthesis System (Life technologies TM. Grand Island, NY). Full-length sequence of rat Omi was created by subcloning the PCR product amplified with primers: 5′-AGC AGA TGG CTG CGC TGA A-3′ and 5′-AGC AAG GAG GAA ATC AGA GCA-3′ (IDT, Coralville, Iowa) using CloneAmp TM HiFi PCR Premix (Clontech Laboratories, Inc. Mountain View, CA). Full-length pcDNA3.1(+) Omi was created by subcloning the full-length Omi PCR product with primers: 5′-ATT AAG CTT ATG GCT GCG CTG AAG-3′ and 5′-TGC GAA TTC TTA TTC AGT TAT TCT GTG AC-3′ into the pcDNA3.1(+) vector at sites HindIII and EcoRI. Full-length pEGFP-N1 omi was created by subcloning the full-length Omi PCR product with primers 5′-ATT AAG CTT ATG GCT GCG CTG AAG-3′ and 5′-TGC GAA TTC AAT TCA GTT ATT CTG TGA C-3′ at sites HindIII and EcoRI.

### Cell culture, transfections, and drug treatments

Commercially available H9C2 rat embryonic cardiac myoblasts and transgenic H9C2 cells overexpressing mitochondrial Omi/HtrA2 were cultured in Dulbecco’s modified eagle medium (DMEM) supplemented with 10% fetal bovine serum (FBS) in a 5% humidified CO2 incubator at 37 °C. Transfection was performed using Lipofectamine 2000 reagent (Invitrogen, USA) according to the manufacturer’s instructions. Mitochondrial Omi/HtrA2-stable H9C2 cell lines were purchased from the Saiye Biotech Limited Company, which were established by limiting dilutions with 400 mg/ml G418 (Gibco), and mitochondrial Omi/HtrA2 was analyzed by western blotting and fluorescence microscopy. At approximately 80% confluence, cells were serum starved for 12 hours in DMEM supplemented with 1% FBS. For hypoxia-reoxygenation (H/R) experiments, hypoxia was induced by cellular incubation in 1% FBS DMEM in an anaerobic chamber equilibrated with 95% N2/5% CO2 at 37 °C for 24 hours. The cells were reoxygenated under normoxic conditions in a 95% air/5% CO2 humidified atmosphere at 37 °C for indicated times. Various doses of Ucf-101 and cyclosporine A were administered to H9C2 cells during the 24 hour period pre-hypoxia.

### Cellular Fractionation

Both cell protein and tissue protein were prepared per standard protocol^9^,^10^, and lysate protein concentrations were determined via the Bradford assay (Bio-Rad, USA). Preparation of mitochondria and cytosolic extracts from tissue was described thoroughly previously. To isolate mitochondrial and cytosolic fractions, cells were subjected to PBS washes and the Mitochondria/Cytosol Fractionate Kit (BioVision, CA). Cells were homogenized with a glass Dounce homogenizer and Teflon. Cytosolic and mitochondrial fractions were separated by differential centrifugation (5 minutes at 1000 g, 30 minutes at 17530 g).

### Western Blot Analysis and Quantitative PCR

Equal quantities of proteins (30–50 μg/lane) were submitted to 15% SDS-PAGE, electro-transferred onto polyvinylidene fluoride membranes, and incubated with primary antibodies against rabbit anti-HtrA2/Omi (Cell Signaling Technology, #2176), rabbit anti-XIAP (Cell Signaling Technology, #2042), rabbit anti-HAX-1 (Millipore, ABT65), rabbit anti-GAPDH (Cell Signaling Technology, #2118), and rabbit anti-Cox IV (Cell Signaling Technology, #4844), mouse anti-EGFP (ab184601). After incubation with the secondary antibodies, protein bands were detected via enhanced chemiluminescence (Pierce, USA). Scanned protein band density was measured by image analysis software. Analysis of gene expression was studied via real time quantitative RT-PCR with SYBR Green (Sigma) detection in the Mx3005 real time-PCR system (Atratagene, USA) as previously described^10^.

### Determination of Myocardial Apoptotic Death and Caspase Protease Activity

Myocardial apoptosis was determined by an *in situ* apoptosis detection kit (Roche, Switzerland), as reported previously^9^,^10^. Briefly, transmural myocardial tissue blocks were fixed in 4% paraformaldehyde in PBS, embedded in paraffin, and subjected to TUNEL staining per manufacturer’s protocol. Apoptotic index was assessed by measuring the percentage of apoptotic nuclei of left ventricular from each slide. The substrates Ac-DEVD-AFC, and Ac-LEHD-AFC were used to determine caspase-3, and caspase-9 activity respectively, following the manufacturer’s guidelines (BIOMOL, USA). Briefly, transmural tissue blocks from left ventricular were homogenized in ice-cold lysis buffer, and centrifuged (12,000 g for 10 minutes at 4 °C). 50 μl of supernatant was then incubated with buffer containing 10 mM dithiothreitol and 5 μl Ac-DEVD/LEHD-AFC (final concentration 200 μM) at 37 °C for 1.5–2 hours. Activity of caspase-3 and 9 was determined by fluorescent microplate reader (BIOTEK, FL-600) at Ex: 400 nm, Em 508 nm. Results were expressed as -fold of the control^42^.

### Detection of Phosphatidylserine Translocation by Annexin V-FITC

Phosphatidylserine externalization, a relatively early event associated with apoptotic onset, was assessed with an Annexin V-FITC Detection Kit (BD Biosciences Pharmingen, USA). Briefly, cells were loaded with a saturating concentration of Annexin V-FITC for 15 minutes at 37 °C. Apoptotic cells were counted by flow cytometry (FCM) on a FACScan (Becton Dickinson, USA).

### *In vivo* Echocardiographic and Hemodynamic Measurements

For echocardiography, mice were anesthetized with 1.5% isoflurane. Two-dimensional echocardiographic views of the mid-ventricular short axis were obtained at the level of the papillary muscle tips below the mitral valve (Vevo 770, Visual Sonic, Canada). Left ventricular fractional shortening (FS) and left ventricular ejection fraction (EF) were calculated as previously described^42^. The diastolic (LVIDd) and systolic (LVIDs) diameters of the left ventricle, and the diastolic and systolic thicknesses of the posterior (LVPWd, LVPWs) and anterior (LVAWd, LVAWs) walls of the LV, were measured by echocardiography.

### Statistical Analysis

All values in the text and figures are presented as mean ± SEM. All data (except Western blot density) were subjected to ANOVA followed by post hoc Bonferroni correction. Western blot densities were analyzed with the Kruskal-Wallis test followed by Dunn’s post hoc test. Probabilities of 0.05 or less were considered statistically significant.

## Additional Information

**How to cite this article**: Wang, K. *et al*. Cardiac Specific Overexpression of Mitochondrial Omi/HtrA2 Induces Myocardial Apoptosis and Cardiac Dysfunction. *Sci. Rep.* **6**, 37927; doi: 10.1038/srep37927 (2016).

**Publisher's note:** Springer Nature remains neutral with regard to jurisdictional claims in published maps and institutional affiliations.

## Supplementary Material

Supplementary Figures
