# Supplementary material for: Cardiac Specific Overexpression of Mitochondrial Omi/HtrA2 Induces Myocardial Apoptosis and Cardiac Dysfunction
Source: Sci Rep. 2016 Dec 7;6:37927. doi: 10.1038/srep37927 (PMC5141441; doi:10.1038/srep37927)
Supplement: Supplementary Figures [file srep37927-s1.pdf]

Cardiac Specific Overexpression of Mitochondrial Omi/HtrA2 Induces Myocardial Apoptosis and Cardiac Dysfunction

Ke Wang[1], Yuexing Yuan[2], Xin Liu[1], Wayne Bond Lau [2], Lin Zuo[3], Xiaoliang Wang [3], Lu Ma[1], Kun Jiao[1], Jianyu Shang[1], Wen Wang[1], Xinliang Ma[1,2,4], Huirong Liu, MD, PhD[1,4*]

[1] Department of Physiology and Pathophysiology, School of Basic Medical Sciences, Capital Medical University, Beijing, 100069, P. R. China.

[2] Department of Emergency Medicine, Thomas Jefferson University, 1020 Sansom Street, Philadelphia, PA 19107, USA.

[3] Department of Physiology, Shanxi Medical University, Taiyuan, Shanxi, 030001, P. R. China.

[4] Beijing Key Laboratory of Metabolic Disturbance Related Cardiovascular Disease, Capital Medical University, Beijing, 100069, P. R. China.

*Address proofs to:

Huirong Liu, MD, PhD

Department of Physiology and Pathophysiology

School of Basic Medical Sciences

Capital Medical University

10 Xitoutiao, You An Men

Beijing

P. R. China, 100069

Tel: 011-86-10-83911830

Fax: 011-86-10-63294027

E-mail: liuhr2000@126.com

Supplemental Figure 1

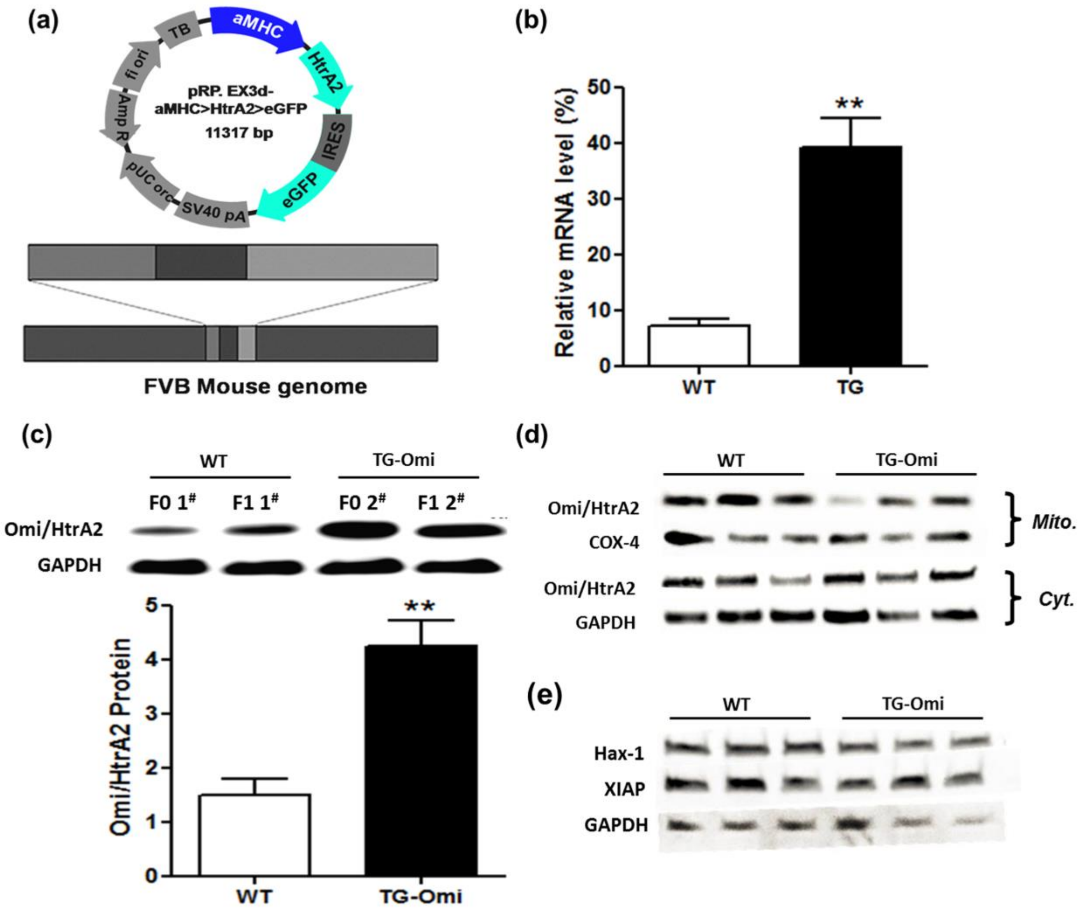

Supplemental Figure 2

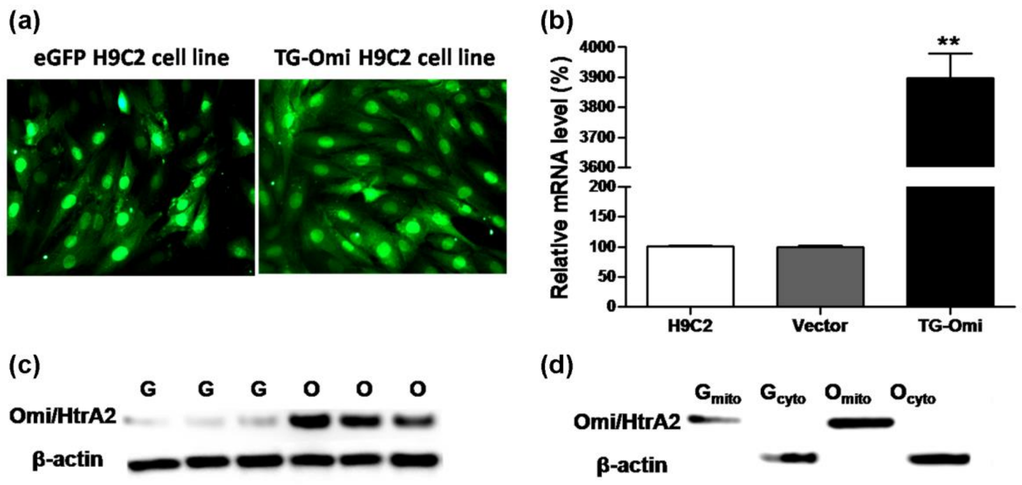

Supplemental Figure Legend

Supplemental Figure 1. General characteristics of cardiac-specific Omi/HtrA2 transgenic mice. **(a)**, Schematic illustration of DNA construct, consisting of cardiac-specific alpha-myosin heavy chain (aMHC) promoter and mouse Omi/HtrA2 cDNA. **(b)**, Omi/HtrA2 mRNA expression was measured by quantitative RT-PCR with mRNA isolated from the left ventricles of WT mice and Omi/HtrA2 transgenic mice (TG - Omi). **(c)**, Western blot of Omi/HtrA2 protein in left ventricle protein extracts from WT mice and Omi/HtrA2 transgenic mice (TG - Omi). **(d)**, Western blot of Omi/HtrA2 protein in either mitochondrial (mito.) or cytosolic (cyto.) fraction from WT mice and Omi/HtrA2 transgenic mice. **(e)**, Western blot of XIAP and HAX-1 protein in left ventricle protein extracts from WT mice and Omi/HtrA2 transgenic mice. n = 3 - 5/genotype. $^{**}p < 0.01$ *vs.* WT

Supplemental Figure 2. General characteristics of H9C2 cell lines stably transfected with mitochondrial Omi/HtrA2. **(a),** Fluorescent images of green shows positive transfected H9C2 cell with Omi/HtrA2. **(b),** Omi/HtrA2 mRNA expression was measured with quantitative RT-PCR with mRNA. **(c)**, Western blot of Omi/HtrA2 protein in whole cell fraction. **(d)**, Western blot of Omi/HtrA2 protein in either mitochondrial or cytosolic fraction. (G: H9C2 cell line stably transfected with eGFP; O: H9C2 cell line with both eGFP and mitochondrial Omi/HtrA2; mito: Mitochondrial fraction; cyto: Cytoplasmic fraction) n=10-12each. $^{**}p < 0.01$ *vs.* eGFP H9C2 cell line (Vector)
